# An efficient visual servo tracker for herd monitoring by UAV

**DOI:** 10.1038/s41598-024-60445-4

**Published:** 2024-05-07

**Authors:** Wei Luo, Guoqing Zhang, Quanqin Shao, Yongxiang Zhao, Dongliang Wang, Xiongyi Zhang, Ke Liu, Xiaoliang Li, Jiandong Liu, Penggang Wang, Lin Li, Guanwu Wang, Fulong Wang, Zhongde Yu

**Affiliations:** 1 North China Institute of Aerospace Engineering, Langfang, 065000 China; 2 Key Laboratory of Land Surface Pattern and Simulation, Institute of Geographic Sciences and Natural Resources Research, Chinese Academy of Sciences, Beijing, 100101 China; 3 Aerospace Remote Sensing Information Processing and Application Collaborative Innovation Center of Hebei Province, Langfang, 065000 China; 4 National Joint Engineering Research Center of Space Remote Sensing Information Application Technology, Langfang, 065000 China; 5 University of Chinese Academy of Sciences, Beijing, 101407 China

**Keywords:** Aerospace engineering, Biodiversity

## Abstract

It is a challenging and meaningful task to carry out UAV-based livestock monitoring in high-altitude (more than 4500 m on average) and cold regions (annual average – 4 °C) on the Qinghai Tibet Plateau. The purpose of artificial intelligence (AI) is to execute automated tasks and to solve practical problems in actual applications by combining the software technology with the hardware carrier to create integrated advanced devices. Only in this way, the maximum value of AI could be realized. In this paper, a real-time tracking system with dynamic target tracking ability is proposed. It is developed based on the tracking-by-detection architecture using YOLOv7 and Deep SORT algorithms for target detection and tracking, respectively. In response to the problems encountered in the tracking process of complex and dense scenes, our work (1) Uses optical flow to compensate the Kalman filter, to solve the problem of mismatch between the target bounding box predicted by the Kalman filter (KF) and the input when the target detection in the current frame is complex, thereby improving the prediction accuracy; (2) Using a low confidence trajectory filtering method to reduce false positive trajectories generated by Deep SORT, thereby mitigating the impact of unreliable detection on target tracking. (3) A visual servo controller has been designed for the Unmanned Aerial Vehicle (UAV) to reduce the impact of rapid movement on tracking and ensure that the target is always within the field of view of the UAV camera, thereby achieving automatic tracking tasks. Finally, the system was tested using Tibetan yaks on the Qinghai Tibet Plateau as tracking targets, and the results showed that the system has real-time multi tracking ability and ideal visual servo effect in complex and dense scenes.

## Introduction

The fourth agricultural revolution, also known as Agriculture 4.0, aims at improving productivity, efficiency, quality, and resilience of agricultural systems, as well as reducing environmental impacts, resource use, and costs^1^. It is a new technology revolution in agriculture supported by policy-makers around the world. It refers to the utilization of advanced technologies including AI, biotechnology, big data, internet of things (IoT), and robotics to achieve sustainable agriculture development^2^. These technologies have been used to improve various aspects of agriculture such as precision farming, smart irrigation, crop monitoring^3,4^ and livestock monitoring^5^. However, how to apply Agricultural 4.0 technology to intelligent monitoring of livestock in high-altitude areas on the plateau is currently a challenge faced by research.

In high-altitude areas of the plateau, due to their complex terrain and harsh natural environment, traditional monitoring methods are difficult to effectively monitor cattle herds, which limits the development of animal husbandry in the plateau area. Traditional animal husbandry usually uses manual operation to identify and track moving herds to obtain real-time information of the herd status, which is a time-consuming, laborious and inefficient method. With the emergence of agriculture 4.0 and the rapid development of the UAV technology, autonomous UAV equipped with embedded computer can conduct real-time tracking and individual differentiation of herds without human intervention^5–7^ to achieve the substantive progress of intelligent grazing.

AI technology can be embedded in intelligent UAVs, providing them with powerful data processing and real-time analysis capabilities, thereby providing strong technical support for intelligent UAVs to monitor cattle herds in livestock farms. AI can assist ranch managers in non-contact and sustainable monitoring and control of cattle herds, enabling them to achieve intelligent management. In addition, it can provide sustainable monitoring and yield prediction for grasslands by analyzing meteorological data, pest and disease conditions, etc. Issuing warnings during the peak period of disease and pest outbreaks helps ranch managers to apply pesticides in a timely manner, ensuring the normal growth of grasslands and the health of livestock. In summary, AI has made significant contributions to the sustainable development of Agriculture 4.0 and has foreseeable application value. However, it is a challenging task to change the tracking targets from individual animals to groups with the help of AI algorithms, and to realize offline real-time monitoring using autonomous UAVs.

Due to the higher flexibility in terms of flight altitude and mission time compared to satellite remote sensing^8^, UAVs show great potential in ecology and conservation zoology research. The development of UAVs provides an efficient and low-cost solution for the application of remote sensing technology in ecology and conservation zoology. It has been drawing increasing attention from ecologists and conservation zoologists in recent years. Especially in wildlife conservation and monitoring, the use of UAV technology can quickly cover large and hard-to-reach areas, reduce human risk and interference, and provide high-resolution wildlife images and videos^9–11^.

In addition, the combination of UAVs and deep learning has been used for a variety of animal studies such as mapping habitats, estimating species abundance and distribution, monitoring individual and group behavior, measuring physiological parameters, assisting in anti-poaching efforts and studying anti-predator responses^12,13^. UAVs can also help dairy patterns, tracking lost cows, and improving safety^14,15^. However, UAVs also have some limitations in monitoring animal. As the camera is mounted to the UAV, the rapid movement of UAV can lead to drastic changes in the view field of the camera, resulting in tracking failures or disappearance of the target object from the field of view. Therefore, the improvement in the motion control method of UAVs becomes very important.

In recent years, deep learning has also been applied to various domains of animal science, such as wildlife ecology, conservation biology, animal behaviour, animal welfare and animal breeding^16–18^. One of the main advantages of deep learning is its ability to automatically extract features and patterns from large and complex datasets, including images, videos, sounds, texts, etc. This can reduce the needs for human intervention, manual annotation and domain-specific knowledge, and simultaneously improve the efficiency and accuracy of data analysis. For example, deep learning can automatically identify, describe and count wildlife in the camera-trap images, which are widely used for monitoring wild animal populations and habitats^17^. Besides, deep learning can also automatically detect and track animal movements and postures in videos, which are valuable information for scientific study of animal behaviour and welfare^18–23^.

Object detection based on deep learning architectures can be categorized into fast detection, shortcut connection and region-based networks. These networks are effective from perspectives of processing speed, accuracy and so on. Therefore, they are widely used for animal farming. Particularly, Single Shot Multi-Box Detector (SSD) and YOLO V3 have the advantage of processing speed, and Regional Convolutional Neural Network (RCNN) is advantageous with respect to the processing speed as well as the accuracy, so that they are the mostly applied networks currently. In relatively simple cases such as optimal lighting and clear view, the combination of different shallow networks (e.g., Visual Geometry Group (VGG) + Convolutional Neural Network (CNN)) might achieve satisfactory performance^24^. However, in complex scenarios such as real commercial environments, to enhance the model capacity for sufficient environmental variations, it is necessary to combine multiple networks, for example, UNet + Inception V4^25^, and VGGNet + SSD^26^. Besides, even for the same model, a parallel combination to create the two-streamed connection could also improve the detection performance^15,27^.

Multi-object tracking (MOT) typically refers to the detection, the recognition, and the tracking of multiple objects (e.g., pedestrians, cars, and animals) in videos without prior knowledge of the target number. Different targets have different IDs to achieve subsequent trajectory prediction, accurate search, and other tasks. In recent years, various MOT methods have been proposed and widely applied, such as monitoring^28^, traffic monitoring^29^, autonomous driving^30^, and animal monitoring^31–33^, aiming at object collision avoidance^34^ or target tracking^35,36^. However, due to the crowded environments and the occluded objects, the results of MOT could be influenced by the difficult problem configuration, which leads to performance limitations in such scenarios. In addition, due to the extensive application of MOT methods, the importance of the MOT is still a challenging subject for the relevant research^37–39^.

In recent years, with the rapid development of deep learning technology, the target detection performance has been significantly improved. With the emergence of the deep learning-based object detectors, tracking through detection has already become the most-focused method in MOT research^39^. This method utilizes the knowledge of object location to establish a model that can associate with objects over time. In recent studies, the algorithm of KF has been used as the motion model to improve the object correlation over time^40–43^. In 2016, SORT was proposed^40^, which applies KF to estimate the object states and associates KF prediction with new object detection using the Hungarian algorithm^44^. One year later, an optimized Deep SORT was proposed by Wojke et al.^43^, which includes a new cascading association procedure using the object appearance characteristics based on CNN. In this data association algorithm, the similarity of the object appearance characteristics and the Mahalanobis distances between the object states are combined, and the SORT data association is used in the later stage of mismatched states. Despite using CNN, high frame rates on target tracking benchmarks were achieved with the Deep SORT approach. Chen et al. proposed an algorithm similar to the Deep SORT, namely MOTDT^41^, which employs a scoring function completely based on CNN to optimally select candidates. The Euclidean distance in the extracted object appearance characteristics is also adopted to optimize the association steps. Recently, He et al.^42^ proposed a GMT-CT algorithm, which combines deep feature learning and graph partitioning. The graph is constructed using extracted object appearance characteristics for association steps to more accurately model the correlation between the measurements and the trajectories.

With the rapid development of autonomous UAVs, the abilities of UAVs for low-speed flying, hovering, laterally flying, and maneuvering in confined spaces make visual servo control a promising platform for performing tasks such as inspection, surveillance, and monitoring. In recent years, various studies have been conducted on the visual servo control of the UAVs, including quadrotors^45,46^, airships^47^ and UAVs^48^. Strategies for navigation and control of UVAs using only vision with feedback loops for monitoring known objects were proposed previously^49^. Stability control methods for quadrotor helicopters using vision as the primary sensor were also reported^50^. In this work, the helicopter attitude is estimated and used for vehicle control. Some studies on vision-based autonomous flight have been reported previously^51–53^. Among the different visual servo control models based on images, adaptive control^54^, Proportion Integration Differentiation (PID) control^55^, sliding model control^56^ and neural network control^57^ have been mainly used to enable one-camera UAVs to explore environments and avoid obstacles. Especially, the PID controller has very wide application in this field due to its high robustness.

A comprehensive review and analysis of existing methods indicate that using UAVs for MOT in complex and dense scenes remains a challenging task in contemporary research. In response to these challenges, this article proposes a UAV MOT system suitable for complex and dense scenes. The main contributions of this study are as follows:

(i) Deep SORT is a multi-target tracking method with competitive advantages. However, when the motion of the object is complex and the detection of the object in the current frame is lost, the bounding box predicted by the KF cannot match the input. To overcome this problem, optical flow is used in this work to compensate the KF, improved the prediction accuracy of the target, thereby enhancing the tracking effect.
(ii) The state-of-the-art tracking-by-detection techniques are still suffering from issues such as a large number of false positive tracks. Therefore, we use a low-confidence trajectory filtering extension in Deep SORT to average detection confidence within the first few frames after initialization. Trajectories with low average confidence are filtered out to reduce false positive trajectories, so as to reduce the impact of unreliable detection on tracking.
(iii) Due to the installation of the camera on the UAV, the rapid movement of the UAV can cause a drastic change in the camera's field of view, resulting in tracking failure or complete disappearance of objects from the field of view. Therefore, this study designed a visual servo controller to keep the target within the field of view of the UAV camera, thereby controlling the UAV to automatically complete tracking tasks.

The contents of this paper are arranged as follows. Section “Materials and methods” describes the area and objects of the study, and introduces the overall framework of this system, including detector, tracker and servo control server. In Sections “Results” and “"Discussion”, experimental results are presented and discussed, respectively. Section “Conclusion” summarizes the conclusions.

## Materials and methods

### Area and objects of study

**Figure 1:**
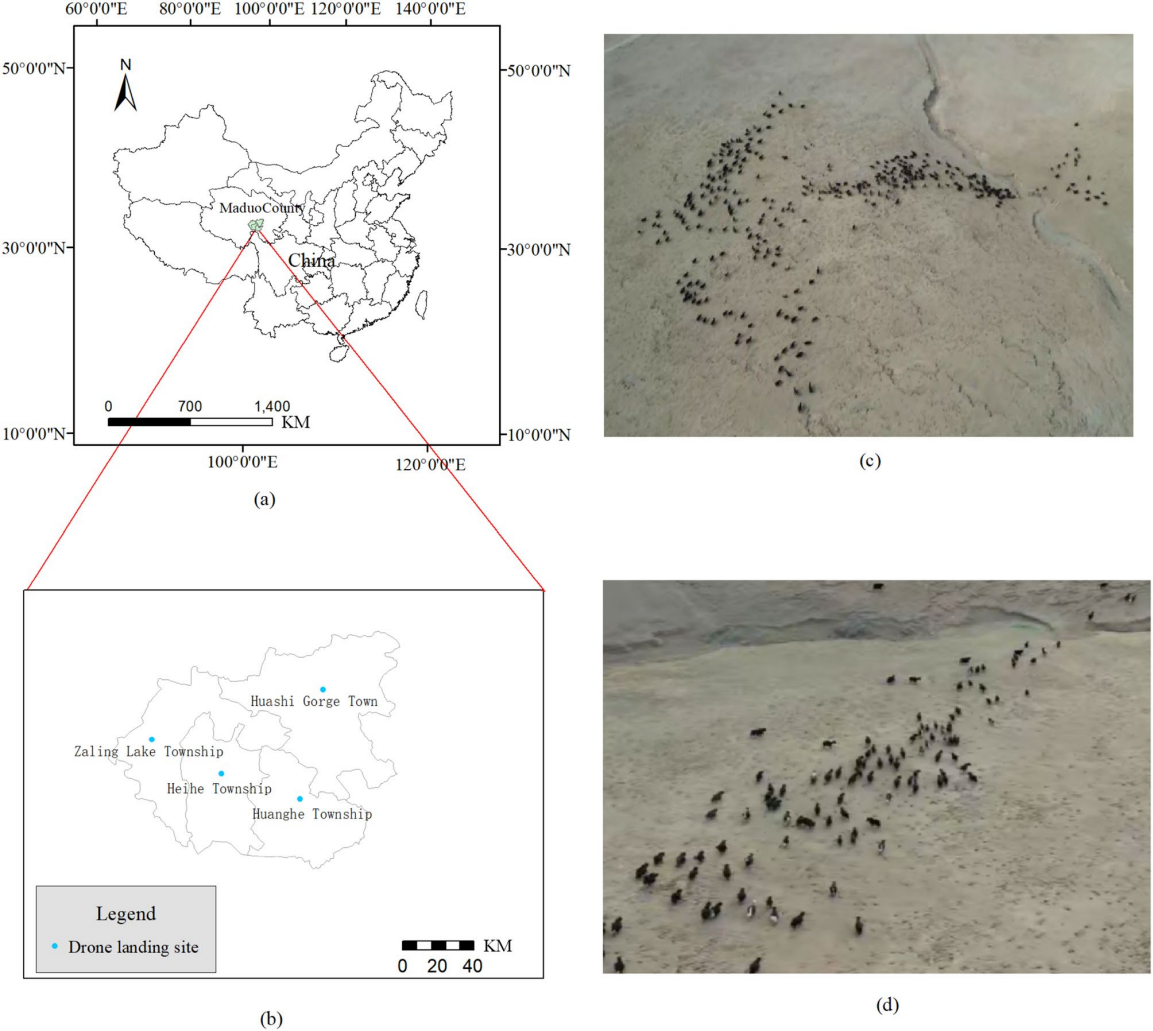
The area selected for the present study: (**a**) The location of Maduo County; (**b**) the distribution of UAV sampling points in Maduo County; (**c**,**d**) aerial images of the studied area.

The area selected for this study is in Maduo County, under the jurisdiction of Golog Tibetan Autonomous Prefecture, in the southern part of Qinghai Province (Fig. 1a). Maduo County locates at the source of the Yellow River and belongs to a typical plateau area, with an average annual temperature of − 4.0 °C. Due to its unique geographical location and ecological environment, the local flora and fauna resources are very abundant, and animal husbandry is particularly developed. It is highly reasonable to choose this area to conduct research on AI-based precise grazing technology.

In April 2023, the authors of this paper and research colleagues went to Maduo County for aerial photography, flying a total of 20 sorties at height of 100 m for sampling. The sampling points are shown Fig. 1b. Finally, the domestic Tibetan yaks were selected as the research objects (Fig. 1c,d), which have a color characteristic of mainly black and gray, and rarely white. The yaks move very slowly and steadily, and their stride frequency is usually between 120 and 140 steps per minute.

### System overview

**Figure 2:**
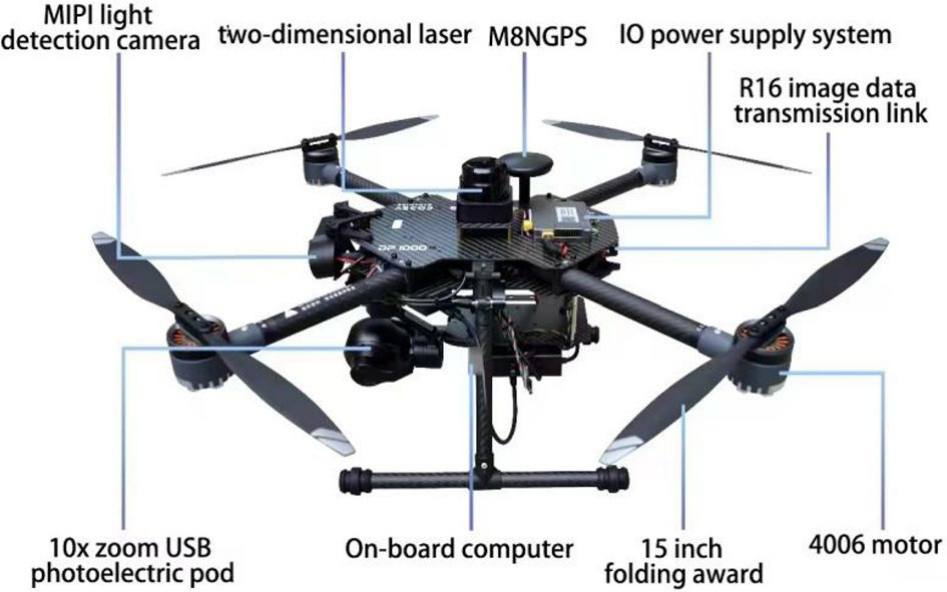
Data acquisition equipment used for this study.

To acquire data in the selected area, a P600 type intelligent UAV (Chengdu Bobei Technology Co., Ltd., China) was used (Fig. 2). The specific parameters of the UAV are detailed in Appendix A to the supplementary material. Compared to other models of intelligent UAVs (such as P230, P450, Dji Phantom, etc.), the P600 UAV has outstanding advantages in flight stability, endurance, and load capacity, making it more suitable for long-term data collection in cold high-altitude areas. It is equipped with an RTK positioning system, with a positioning accuracy of up to centimeters, a more precise flight path, and a more stable attitude. It can collect high-quality data in complex high-altitude areas and fly safely. The body is equipped with an NX onboard computer with a computing power of up to 21TOPS, which can run most mainstream algorithms and perform real-time data processing and analysis while collecting data.

In addition, it is equipped with pods, two-dimensional planar Lidar, GPS and other intelligent devices, to achieve pod selection and tracking, LiDAR obstacle avoidance, as well as UAV position and speed guidance flight. Furthermore, Q10F 10 × single light pod equipped with a USB interface was incorporated with the P600 UAV, and a specific robot operating system (ROS2) driver was developed for P600. This equipment is able to capture real-time images through the pod within the airborne computer. It could also follow the targets and adjust the position to always keep a constant distance from moving targets. During the target tracking process, both UAV and pod can achieve fully autonomous control via ROS2.

The Q10F 10 × single light pod can obtain real-time images of targets with an image resolution of up to 5 cm, providing target data for the built-in tracking and detection algorithms of ROS2. The ROS2 system can enable the P600 UAV to obtain real-time images of targets from the onboard computer through the Q10F 10 × single light pod. Then, through the built-in tracking algorithm in the onboard system, based on image vision, it can not only recognize and track specific targets (targets and UAVs), but also calculate the approximate distance between the UAV and the tracked target by changing the size of the target detection box in the vision. In addition, the ROS2 system can also adjust the UAV's position as the target approaches, always maintaining a fixed distance from the target to avoid interfering with the target's activities. The combination of the Q10F 10 × optronic pod and the ROS2 system allows the P600 UAV to not only be fully autonomous, but also to track the target with an intelligent pod.

**Figure 3:**
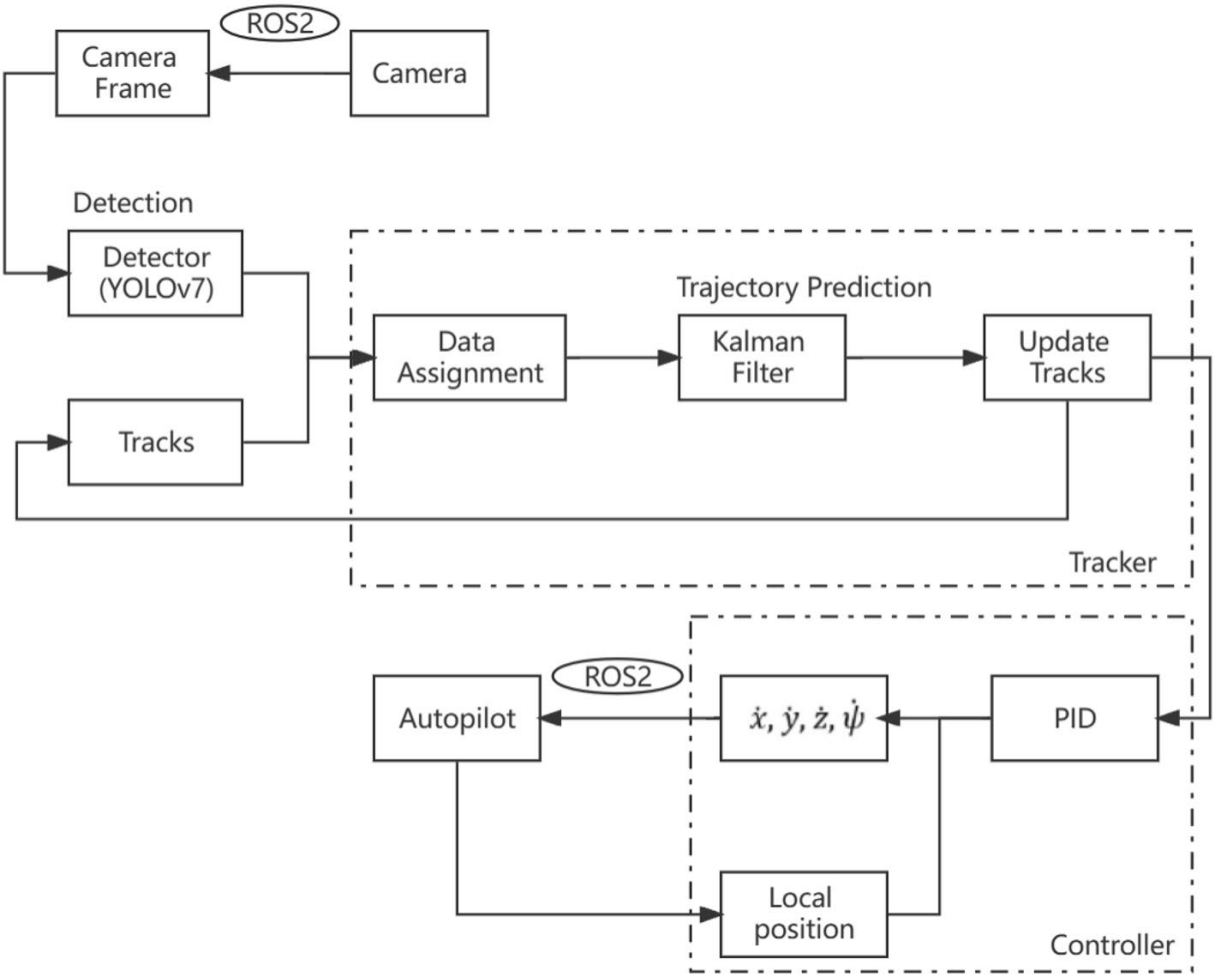
The overall technical framework proposed in this study.

Based on the function, the system can be divided into three components, including the controller, the detector, and the tracker. The overall technical route is shown in Fig. 3. In this study, two walking Tibetan yaks were selected as the tracking objectives. When each of the camera frames processes, several confirmed tracking paths are sent to the control system, which calculates the required speed in 4 different control variables in accordance with the embedded algorithms and the real-time location of UAV. Afterwards, the speed is sent to the autopilot to control UAV for tracking the targets. As a basic component of the control system, ROS2 plays a crucial role in information exchange between UAV and the tracking program. Moreover, the algorithms for speed calculation in 4 control variables differ from each other, so that they are described separately in Section “Improved Deep SORT algorithm”.

### Detector

Since this study aims at tracking and identifying target objects in scenarios with high dynamic density and low training data, YOLOv7^58^ was chosen as the baseline model for balancing the limited computational power and the airborne computer speed.

The YOLOv7 model was developed in 2022 by Wang and Bochkovskiy et al., integrating strategies including E-ELAN (Extended Efficient Layer Aggregation Network)^23^, cascade-based model scaling^59^ and model reparameterization^60^ to appropriately balance the detection efficiency and accuracy. The YOLOv 7 network comprises 4 different modules: Input module, backbone network, head network, and prediction network.

The main reason for choosing YOLOv7 as the detection model is that current deep learning based object detection algorithms can be divided into two-stage detection methods and single-stage detection methods. The two-stage detection methods include RCNN, Fast-RCNN, Mask RCNN, etc. Single stage detection methods include SSD, YOLOv1-YOLOv8, etc. Compared to two-stage detection methods, single-stage detection methods have better real-time performance and are more suitable for UAV platforms. In the single-stage detection method, compared with SSD and YOLOv1-YOLOv6 models, YOLOv7 performs better in terms of comprehensive detection accuracy, detection rate, and network convergence speed. Compared to the YOLOv8 model, although YOLOv7 may not perform as well as YOLOv8 in terms of detection speed and accuracy, it is lighter in model complexity and can be deployed on unmanned aerial vehicle platforms with limited computing power.

We selected 20 domestic yak video sequence data from the study area as datasets, 10 of which were used as target detection datasets and 10 as target tracking datasets. These two sets of data were used as two benchmarks for yak detection and tracking, and YOLOv7 model detection was used to identify yaks.

To improve the model's ability to detect yaks, the target detection dataset was further divided into 3 sets, including training, validation and test sets with a ratio of 7:2:1. The YOLOv7 model is adopted to train the dataset by adjusting the model parameters to achieve high stability of the model. The yak hair color is generally pure black or black and white. In order to obtain more yak hair texture features, 2400 yak hair images from 10 video sequences in the target detection dataset were intercepted and the dataset was divided with the ratio of 7:2:1, which was trained again using YOLOv7.

### Tracker

**Figure 4:**
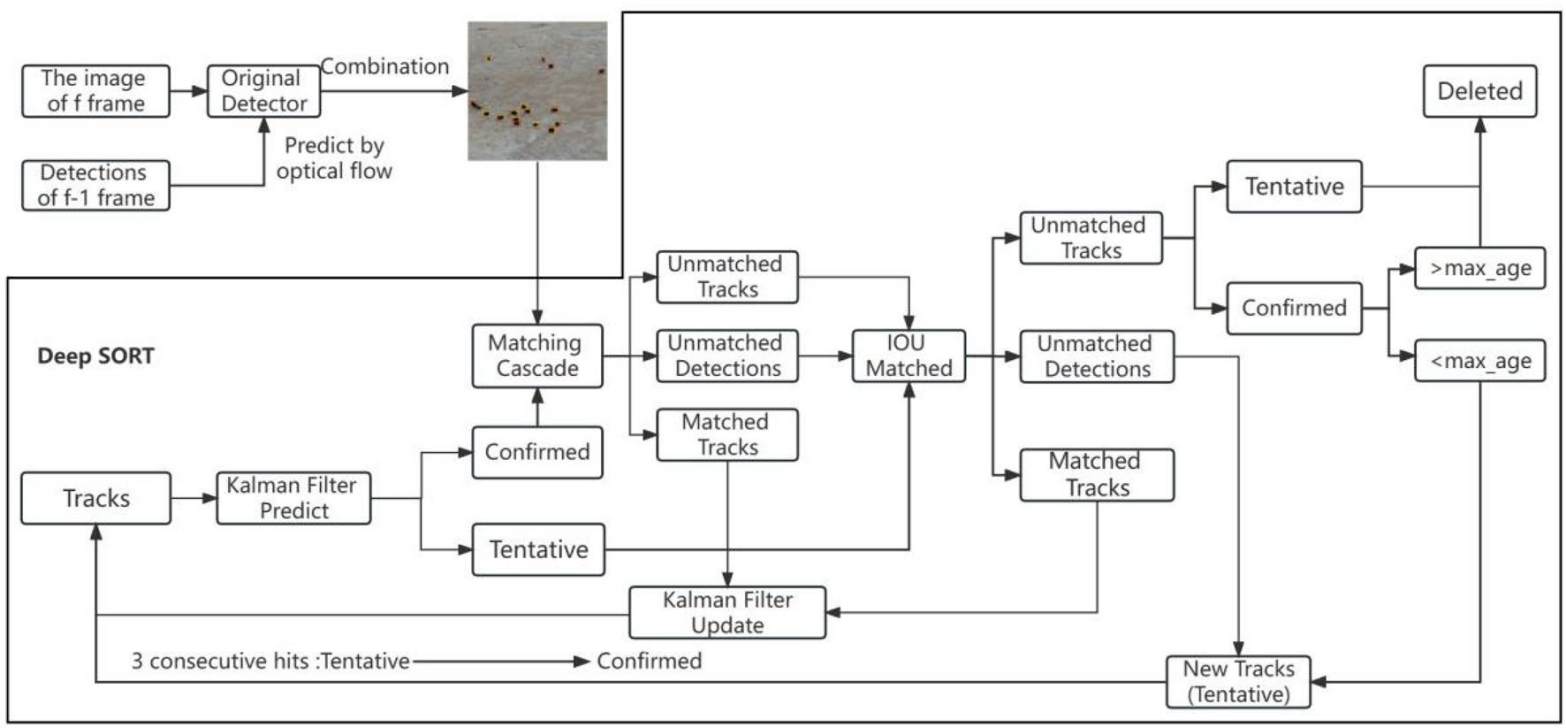
Multiple object tracking pipeline.

The Deep SORT algorithm was used as the baseline algorithm for the tracker and two improvements in the algorithm were made. Firstly, optical flow for motion estimation^7^ was introduced into the scheme to improve the motion prediction accuracy of KF. Secondly, an extended version of the original tracking method, named as low confidence track filtering method, was used to improve the ability of the tracker for handling unreliable detection results, which might occur in the real-world target detection due to the complex environment. By this means, the quantity of the false positive paths could be significantly reduced, avoiding the unreliable detection. The specific process is shown in Fig. 4.

In order to apply Deep SORT to yak tracking and monitoring, we first need a large number of yak datasets to extract the appearance features of trained yaks. Since the target tracking dataset has 10 video sequence data, which is insufficient, we re-generate the target tracking dataset by setting the truncation rate and occlusion rate parameters to cut, rotate and synthesize the video frame images.

The occlusion rate defines the degree of occlusion by the proportion of the yak bounding box that is occluded. We categorize the degree of occlusion into three categories: no occlusion, partial occlusion, and heavy occlusion. Specifically, a yak is defined as partially shaded if it is between 1 and 50% shaded, and as heavily shaded if it is greater than 50% shaded.

The cutoff rate is an indication of how far the yak is outside the bounding box and is used for training sample selection. In order to minimize the effect of noise, we discarded yak data with truncation rate more than 0.5 or occlusion rate more than 0.5. About 100 video data with the same interval were selected as a batch, and the video frames were intercepted and then resized to JPEG images of the same size (500,500) to obtain a total of 6000 yak images. We annotated the 6000 images using Label-image software and stored them in XML format as a target tracking dataset to be used as a benchmark for yak tracking.

The Deep SORT algorithm adopted in this work uses KF to estimate the existing track in the current frame. The states applied in KF are defined as $(x,y,\gamma ,h,\dot{x},\dot{y},\dot{\gamma },\dot{h})$, in which $(x,y,\gamma ,h)$ represents the bounding box position, and $(\dot{x},\dot{y},\dot{\gamma },\dot{h})$ represents the single coordinate velocity. KF involved in Deep SORT is the standard version using a constant velocity and a linear observation. When each new frame appears, the position of each existing track will be estimated based on the previous one, and the track estimation only needs spatial information.

In order to achieve the appearance information of the detection results and tracks, appearance descriptors were used for extracting features from the detection images and tracking the images from the previous frames. As a CNN model trained on a large-scale recognition dataset, the appearance descriptor is capable of extracting features in the feature space based on that the features from same identity are similar to each other.

By estimating the position and appearance information of existing tracks, in each future frame new detection results could be associated with the existing tracks. New detection results need to have confidence levels above the detection confidence threshold $t_{d}$ to become candidates for data association. All the detections do not meet this criterion will be filtered out. A cost matrix is used in Deep SORT for representing spatial and visual similarity between the new detections and the existing tracks, which contains two distance parameters. The first one is the Mahalanobis distance represented by formula (1) for spatial information:

1 $$ d^{(1)} (i,j) = (d_{j} - y_{i} )^{T} s_{i}^{ - 1} (d_{j} - y_{i} ) $$

where $y_{i}$ represents the i-th orbit, $s_{i}^{ - 1}$ represents the covariance of d and y, $(y_{i} ,s{}_{i})$ represents the projection of the i-th orbit in the space of measurement, and $d_{j}$ represents the j-th new detection. It is the distance between the estimated position of the i-th orbit and the j-th new detection. The second distance represents the appearance information as shown below by formula (2):

2 $$ d^{(2)} (i,j) = \min \left\{ {1 - r_{j}^{T} r_{k}^{(i)} |r_{k}^{(i)} \in R_{i} } \right\} $$

where r represents an appearance descriptor, $R_{i}$ represents the appearance of the last one hundred objects associated to the i-th track. Besides, each of the distance is accompanied by gate matrix $b_{i,j}^{(1)}$ and $b_{i,j}^{(2)}$, if the distance is less than a predefined threshold, it is equal to 1, otherwise it is equal to 0. The comprehensive cost matrix is presented in formula (3):

3 $$ c_{i,j} = \lambda d^{(1)} (i,j) + (1 - \lambda )d^{(2)} (i,j) $$

The gate function $b_{i,j} = \prod {_{m = 1}^{2} b_{i,j}^{(m)} }$ is used to set the threshold, it is equal to 1 only when both the space and the appearance gate functions are 1, otherwise, it is equal to 0, indicating whether (i, j) effectively matches both space and appearance. The cost matrix is used for each of the new frame to associate the new detection with the tracks of the existing gate matrix.

In case of a successful association of the new detection with the existing track, the new detection is included into the track, and track shows a non-association age of zero. In case the new detection cannot be associated with the existing track in the F-frame, it is initialized as a tentative track. The original algorithm of Deep SORT verifies whether the tentative track is associated to the new detection in the frame $(f + 1),(f + 2),\;...\;(f + t_{tentative} )$. In case of a successful association, an update of the track to a confirmed one will be conducted. Otherwise, the temporary track will be immediately deleted. For existing tracks without successful association with the new detection in each frame, their non-association ages increase by 1. In case that the non-association ages exceed the threshold, the corresponding tracks will also be removed.

### Improved deep SORT algorithm

#### Combination of KF and optical flow

**Figure 5:**
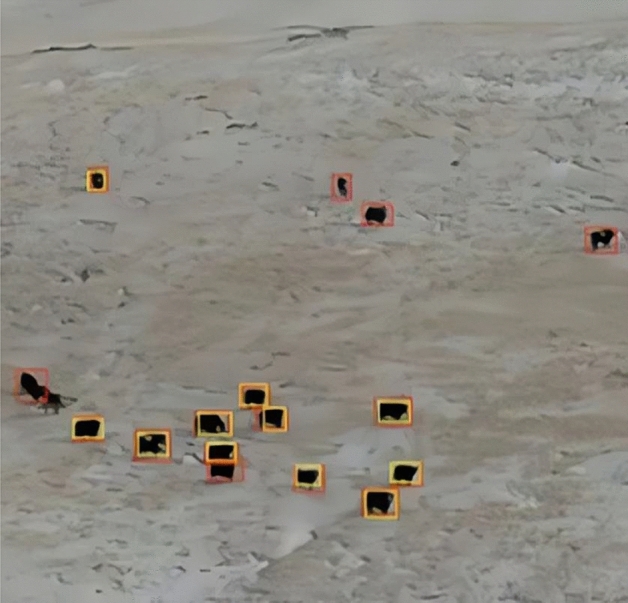
Comparison of the detection results (yellow bounding boxes: Original detection results; red bounding boxes: Detection results from optical flow).

As a classic tracking algorithm, the Lucas-Kanad (LK) optical flow^61^ algorithm has been widely applied due to its competitive real-time speed and strong robustness. To address the problems derived from KF, optical flow is also used to estimate objects in this study, and several assumptions are made, including constant brightness between the adjacent frames, slow movement of the targets, and similar motion pixels of the same images. There is no doubt that the loss of the object detection will challenge the updating of KF and lead to the interruption of trajectory. Therefore, the boundary frames of objects are predicted by using the light flow. In addition to the bounding frame of the F-frame generated with original detector in the data set, optical flow is also adopted to predict the position of the object based upon information in the previous frame. It could provide more historical clues to the information of the previous frame. As shown in Fig. 5, the yellow-colored bounding boxes represent the original detection results and the red ones are the results of the optical flow.

It can be observed that the former produces a more accurate trace input, nevertheless, the primitive detection in complex environments cannot be ignored. To compensate for the adverse effect on performance, combination of them as input for current frame tracking is required, which could provide more reliable state of motion for KF. At the same time, a constant velocity of the object in the frame is assumed, and KF is used to construct a model of linear motion defined in 8-dimensional space:

4 $$ S = (x,y,\gamma ,h,\dot{x},\dot{y},\dot{\gamma },\dot{h}) $$

where (x, y) represent bounding box center coordinates, $\gamma$ represents the aspect ratio, h means high, and $(\dot{x},\dot{y},\dot{\gamma },\dot{h})$ represents the speed of objects in the frame.

#### Filtering of low confidence tracks

False positive tracks derived from unreliable detection results seriously affect the performance of the tracker. At present, the most advanced detection tracking technology still faces a large number of false positive tracks and other problems. To better solve this problem, a filter for low confidence tracks was included into our tracker.

In this tracker, not only a confidence threshold $t_{d}$ is used to filter out detections with confidence below this threshold, but also average confidence values $t_{{ave_{d} }}$ are calculated for new detections in the frame $(f + 1),(f + 2),\;...\;(f + t_{tentative} )$ related to tentative tracks. Only when these average values are greater than the predefined threshold $t_{{ave_{d} }}$, update of the corresponding tentative tracks to the confirmed tracks could be performed. Otherwise, these tentative tracks will be deleted. By this means, the detection results are filtered by two threshold stages of $t_{d}$ and $t_{{ave_{d} }}$ rather than simply by $t_{d}$ alone. Therefore, the threshold $t_{d}$ with a preset lower value can avoid losing detection, and extraction helps for suppressing false positive tracks produced with low confidence threshold (low $t_{d}$). The algorithm used in this study to filter low confidence tracks is detailed in Appendix B to the supplementary material.

### Visual servo control

In this study, a servo control system using helicopters and cameras^62^ is applied for MOT. The system consists of 4 control variables, including lateral control, longitudinal control, vertical control, as well as yaw rate control.

The lateral control aims at keeping the camera frame center aligning with the horizontal middle of tracked objects by using a PID controller that takes the sum of the horizontal distances of each object as the proportional input, the sum of the differences between the current and previous centers as the derivative input, and the cumulative error as the integral input.

According to the PID formula, the lateral speed of $\dot{x}_{uav}$ in the lateral coordinate system of the UAV could be calculated as follows:

5 $$ \dot{x}_{UAV} = Kp_{x} Sp_{x} + Ki_{x} Si_{x} + Kd_{x} Sd_{x} $$

The longitudinal control adjusts the forward and backward speed of the helicopter based on the heights of bounding boxes of the objects, which indicate the distance of objects to the camera. This control unit uses a PID controller that takes the sum of differences between current and minimum heights and between current and maximum heights as the proportional input for calculation of forward and backward speeds, respectively. Besides, it takes the sum of height change rates of each object as the derivative input.

The speed required for vertical control of the UAV is divided into two parts: one based on the height of the object, and the other based on the area where the object is located. Therefore, the final velocity $\dot{y}_{uav}$ of the UAV coordinate system is calculated longitudinally by formula (6).

6 $$ \begin{aligned} \dot{y}_{UAV} & = \dot{y}h_{UAV} + \dot{y}a_{UAV} = Kp_{y} Sp_{y} \_a + Kd_{y} Sd_{y} \_a + Kp_{y} Sp_{y} \_h_{b} \\ & + b_{f} Kp_{y} Sp_{y} \_h_{f} + Kd_{y} Sd_{y} \_h \\ \end{aligned} $$

The vertical control loosely regulates the height of the UAV based on a predefined range. In comparison with the response to the lateral speeds, the response of the autopilot to low vertical speeds to achieve accurate height adjustment is relatively slower. Therefore, it is often that after the autopilot receives such a vertical speed command, the height of UAV does not change.

The yaw rate control rotates the helicopter around its vertical axis to keep it perpendicular to the line connecting the two objects outermost of the camera frame, which estimate the yaw angle by using a ratio between horizontal distance and image width, and a ratio between height difference and standard height for each class of objects. Afterwards, this angle is divided by the processing time and multiplied by a coefficient to achieve the yaw rate.

Since the final command is the yaw rate, the calculated yaw rate is divided by the processing time and multiplied by a factor as shown in Eq. (7).

7 $$ \dot{\phi }_{UAV} = Kp_{\phi } \frac{{\phi_{UAV} }}{\Delta t} $$

In summary, after introducing the principle for calculation of the required speeds in all 4 directions, the complete equation of the final speed command based on the world transformation Eqs. (5), (6) and (7) is as follows:

8 $$ \begin{aligned} V_{world} = & \left[ {\begin{array}{*{20}c} {\dot{x}} \\ {\dot{y}} \\ {\dot{z}} \\ {\dot{\psi }} \\ \end{array} } \right]_{World} = \left[ {\begin{array}{*{20}c} {\cos \psi } & { - \sin \psi } & 0 & 0 \\ {\sin \psi } & {\cos \psi } & 0 & 0 \\ 0 & 0 & 1 & 0 \\ 0 & 0 & 0 & 1 \\ \end{array} } \right] \\ & \left[ {\begin{array}{*{20}c} {Kp_{x} Sp_{x} + Ki_{x} Si_{x} + Kd_{x} Sd_{x} } \\ \begin{gathered} Kp_{y} Sp_{y} \_a + Kd_{y} Sd_{y} \_a + Kp_{y} Sp_{y} \_h_{b} \hfill \\ + b_{f} Kd_{y} Sd_{y} \_h_{f} + Kd_{y} Sd_{y} \_h \hfill \\ \end{gathered} \\ {Kp_{z} \Delta h} \\ {Kp_{\psi } \frac{\psi }{\Delta t}} \\ \end{array} } \right]_{UAV} \\ \end{aligned} $$

The flight controller can calculate the expected acceleration (that is, the three-axis expected thrust) according to $V_{World}$ (expected velocity) and the current velocity, and then convert the desired attitude angle according to the UAV dynamics model. The highly dynamic control algorithm of the UAV attitude loop can ensure the speed and stability of attitude tracking.

## Results

The intelligent unmanned field platform embedded with Jetson AGX Xavier launched by NVIDIA^6^ was used for onboard image processing in the experiments. This modular supercomputer has a 512 CUDA-core NVIDIA Volta GPU with a 8-core ARMv8.2 CPU and strong power of AI computation. It shows a 10 times higher power consumption ratio and a 20 times higher performance compared to the previous Jetson TX2 platform (256 CUDA-core NVIDIA Pascal GUP with a CPU of quad-core ARM).

### Metrics for tracking

To objectively compare the performance of different trackers, the experimental results from this study were evaluated based on the metrics defined in the CLEAR MOT metrics:

- PR-MOTA: Under different confidence thresholds, the values of precision and recall are obtained separately, and then the corresponding PR-MOTA can be obtained based on the different precision and recall. MOTA is the multi-target tracking accuracy , a key score for evaluating the tracking performance. It is composed of 3 calculation errors, including false positive (FP), lost target (FN), and identity switch (IDs). It measures the performance of the tracker in detecting targets and maintaining trajectories, independent of the accuracy of the target location estimation.
- PR-MOTP: It is derived from the values of precision and recall under different confidence thresholds. MOTP is the multi-target tracking precision, which is a measure of the tracker's ability to estimate the target position.
- PR-MT: It is originated from the values of precision and recall for different confidence thresholds. MT is the number of primary tracking traces that are successfully tracked during at least 80% of the target’s lifetime.
- PR-ML: It is derived from the values of precision and recall under different confidence thresholds. ML is the quantity of the mostly lost tracks that are not successfully tracked during minimum 20% of the target's lifetime.
- PR-FP: It is the total quantity of FPs.
- PR-FN: It means total quantity of FNs (target not met).
- PR-FM: With different confidence thresholds, the PR-FM is derived from the values of precision and recall. FM is the times of interruption for a track due to missing detection.
- PR-IDSw: It is found under different confidence thresholds based on the values of precision and recall. IDSw, also known as IDs, is the times of the IDs switch for the same target due to misjudgment of the tracking algorithm. The ideal IDs in the tracking algorithm should be 0. It is the total number of identity switches.

### Evaluation of benchmarks

**Figure 6:**
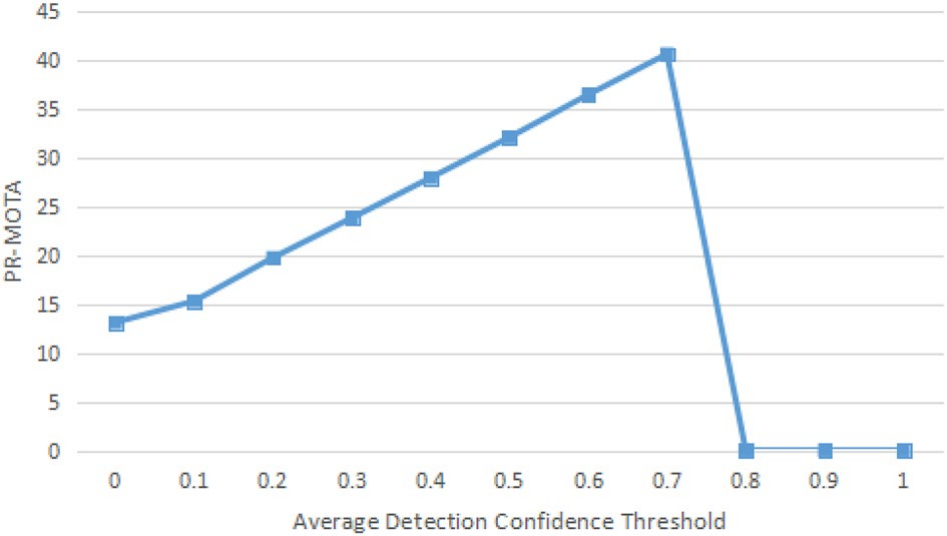
Comparison of the MOTA values of tracking results for 10 training sequences using YOLOV7 detection method under different average detection confidence thresholds.

The resulting mean detection confidence threshold was chosen experimentally. 10 sequences were selected from the target-tracking dataset that were filmed in a relatively more complex environment. It was found that $t_{{ave_{d} }}$ = 0.0 ~ 1.0 for these 10 sequences. The tracker in this study used the YOLOV7 detection method as detection input. The 10 sequences were tested, and the final tracking results were evaluated. Figure 6 shows a comparison of these results.

As shown in Fig. 6, MOTA is better in the presence of $t_{{ave_{d} }}$ (not equal to 0) than in the absence of $t_{{ave_{d} }}$ (equal to 0). The tracking accuracy of the YOLOV7 method keeps improving until $t_{{ave_{d} }}$ reaches 0.7. Therefore, $t_{{ave_{d} }}$ = 0.7 was selected for the experiment of the tracker on the YOLOV7 detection results. It is worth noting that for this detection method, the tracker does not work when $t_{{ave_{d} }}$ is greater than 0.7, because all trajectories are filtered out under that condition.

**Figure 7:**
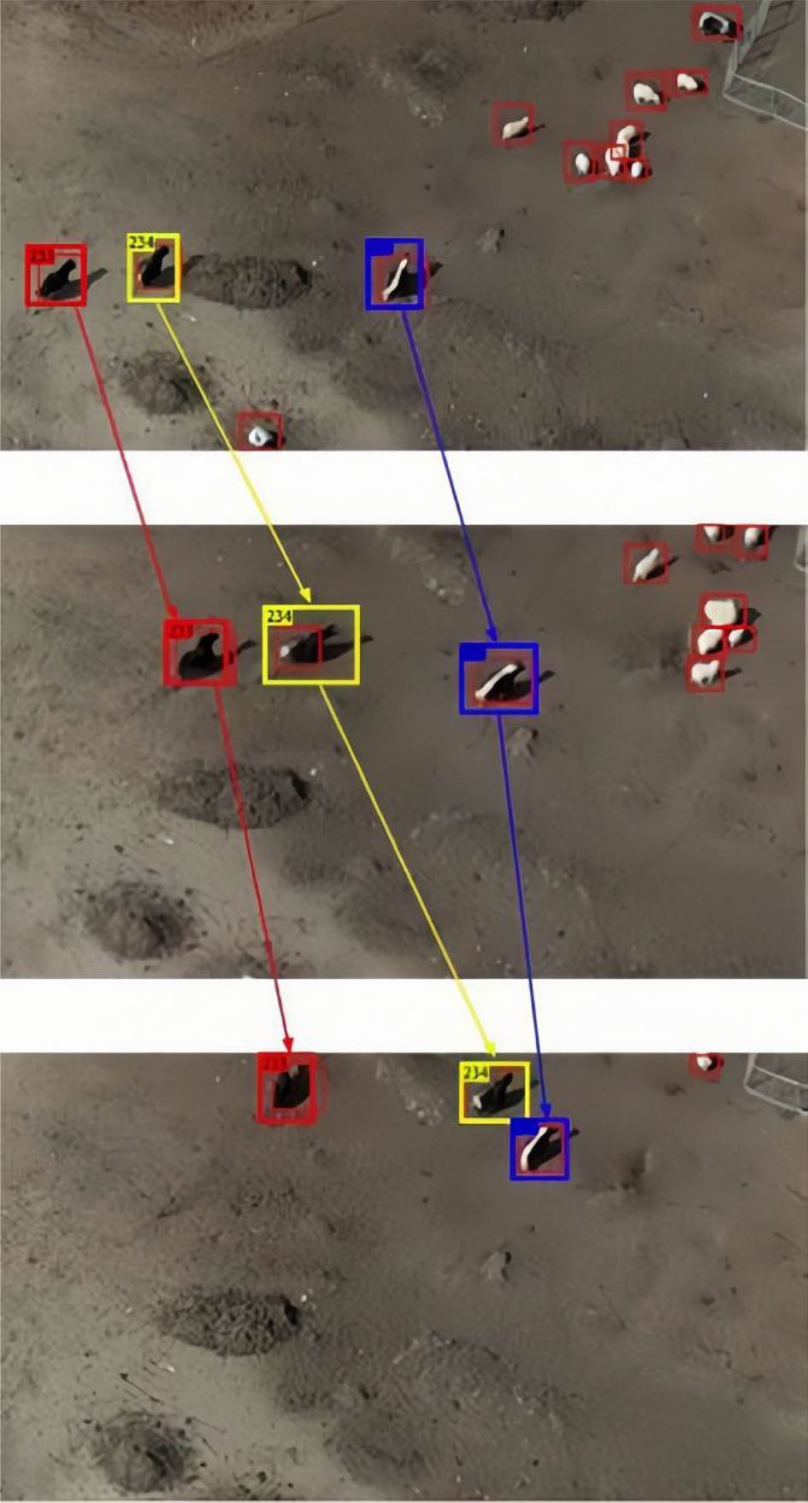
The tracking results on the self-made training sequence “Tibetanyak2023042607” using YOLOv7 detections (td = 0.0 and taved = 0.7).

The tracking results obtained with the proposed tracker in the train sequence "Tibetanyak2023042607" of the target tracking dataset are shown in Fig. 7. This is the result of tracking using the YOLOv7 detection, where the $t_{d}$ and $t_{{ave_{d} }}$ thresholds were set to 0 and 0.7, respectively.

It can be seen from Fig. 7 that there are many red boxes without the identification label. They are the false positive detection filtered out by the filtering algorithm for low confidence tracking. The experimental results show that when $t_{{ave_{d} }}$ = 0.7, the MOTA using the YOLOv7 detection method reaches the optimal value. Tracks with lower average detection confidence in the initial few frames will be deleted. Therefore, the detection confidence $t_{d}$ can be set to a lower value or even zero, to avoid missing detection. When $t_{d}$ = 0.0, many red bounding boxes appear, however, they do not affect the final result of tracking.

The proposed method was tested on an overall test dataset of the target tracks containing ten sequences. As described above, the YOLOv7 tracking results were used as the test input. This detector was chosen for this study because it is the baseline detection method used by most of other trackers, and it exhibits a general good performance. Table 1 shows a comparison of the proposed tracker and state-of-the-art ones with respect to the tracking performance. All these trackers were divided into batch class and online class. In the batch trackers, both the previous and the future information are used for generating tracks in the current frame, while in the online trackers, only the previous information is applied for generating tracks.

**Table 1:** Comparison of the results from the proposed method and other methods (tests conducted on the self-made training date set).

Tracker	Detector	Method	PR-MOTA	PR-MOTP	PR-MT	PR-ML	PR-FM	PR-FP	PR-FN	PR-IDs
IOU^66^	R-CNN^63^	Batch	18.3%	41.9%	14.3%	20.6%	523	2313.5	19,845.1	513
IOU^66^	Comp ACT^65^	Batch	18.4%	41.3%	14.7%	20.1%	379	2459.2	17,125.6	245
IOU^66^	EB^64^	Batch	23.5%	33.2%	17.5%	16..7	248	1456.6	17,054.4	233
IOU^66^	YOLOv7^58^	Batch	33.8%	40.2%	34.6%	19.4%	88	1731.5	17,945.5	70
MOTDT^41^	EB^64^	Online	21.2%	45.6%	18.0%	17.2%	202	3815.8	16,565.2	206
MOTDT^41^	YOLOv7^58^	Online	31.6%	39.3%	33.6%	18.8%	148	7652.4	16,123.5	279
GMT-CT^42^	EB^64^	Online	22.3%	45.5%	18.5%	17.1%	206	5653.2	16,683.2	158
GMT-CT^42^	YOLOv7^58^	Online	32.2%	39.6%	34.6%	18.1%	162	9653.7	16,253.2	186
Deep SORT^43^	EB^64^	Online	20.6%	45.3%	18.1%	17.2%	201	3501.9	16,874.5	180
Ours	EB^64^	Online	22.9%	45.3%	17.8%	17.3%	205	2009.7	17,012.4	166
Deep SORT^43^	YOLOv7^58^	Online	30.4%	39.1%	34.3%	18.5%	159	6456.6	16,456.7	245
Ours	YOLOv7^58^	Online	33.6%	39.2%	32.9%	19.7%	126	2013.1	17,913.2	198

### Validation in actual scenarios

**Figure 8:**
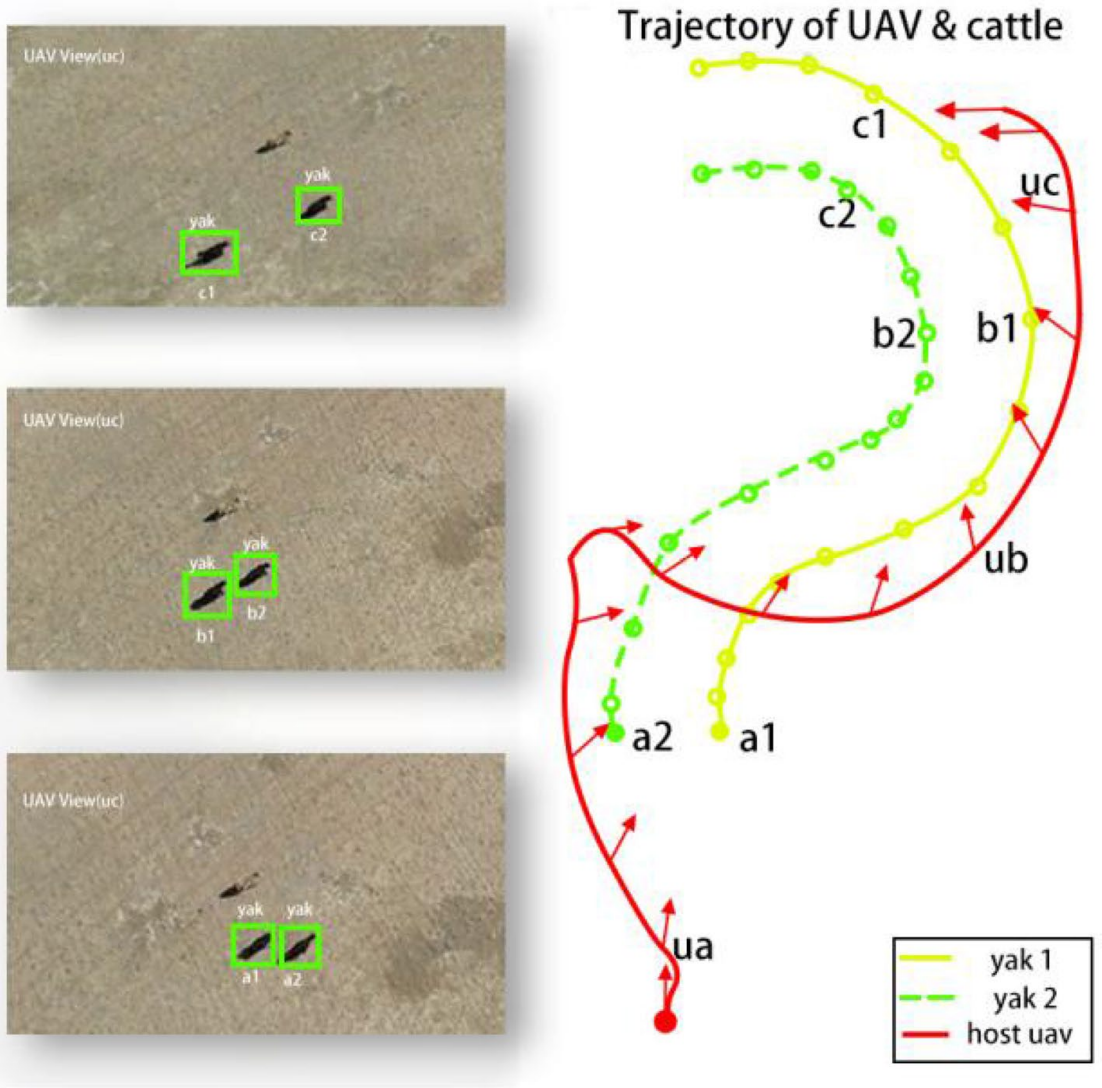
The trajectories of the UAV (red line) and the yaks (yellow and green lines). The left 3 images show the UAV views at the corresponding positions of ua, ub and uc.

In this study, visual servo controller was used to control parameters from four aspects, i. e., lateral, longitudinal, vertical, and yaw rate controls, to assist P600 intelligent UAV flight, and to track and identify multiple yaks. To simultaneously test the comprehensive performances of the visual servo controller in all directions, an experiment with relatively complex object trajectories was designed. In this experiment, a pure black yak and a yak with black and white color were chosen as target objects to verify the tracking ability of the UAV with the visual servo controller. The two yaks walked along concentric arcs of different radii. No overlapped trajectories of yaks and UAVs were observed. Figure 8 shows the relevant trajectories in real scenes.

**Figure 9:**
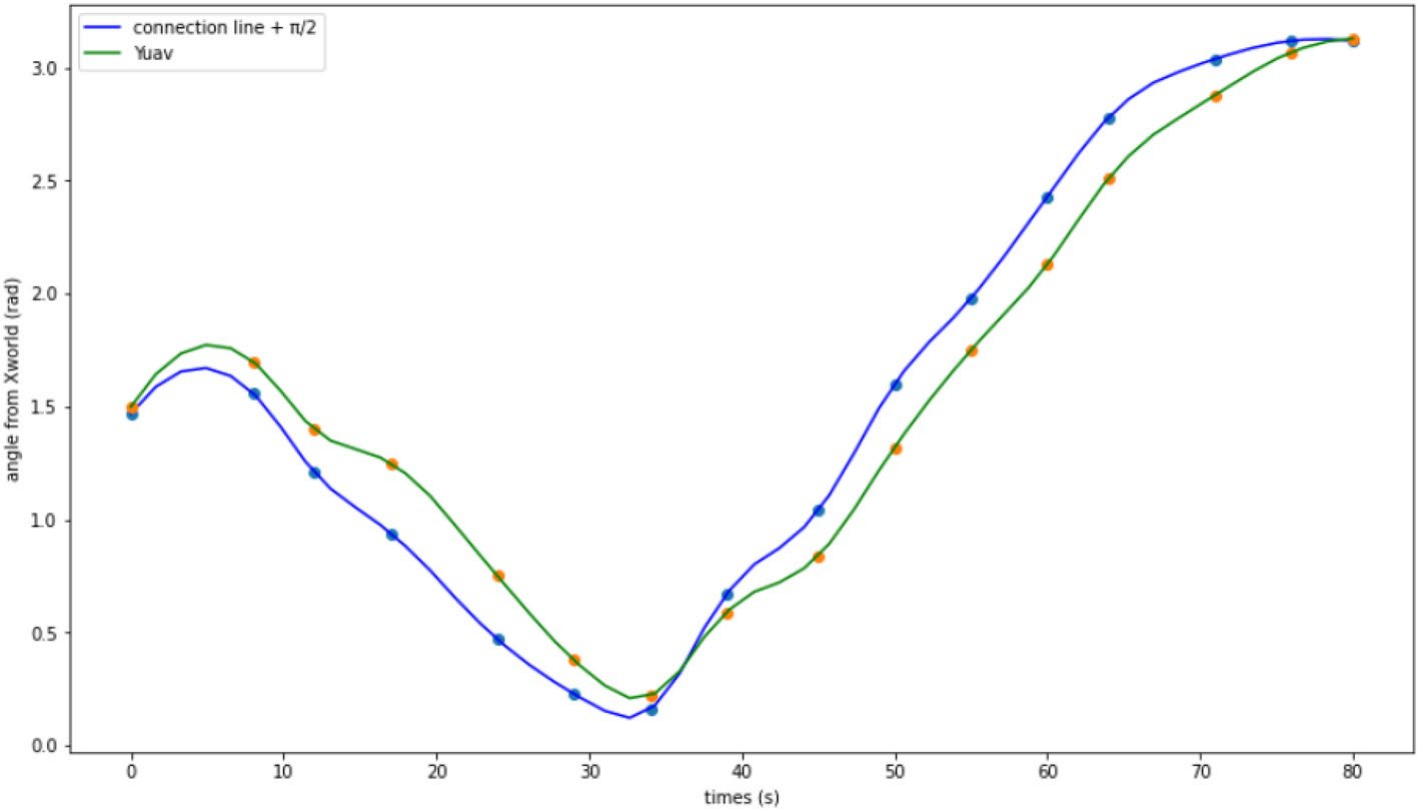
Angle between two object connectors plus Π/2 and Xworld and the angle curve between YUAV and Xworld.

Aiming at further verifying the yaw rate performance of the visual servo controller, the starting points of the trajectories were marked with solid points of the corresponding colors, just similar to the trajectories in Fig. 8. Fifteen arrows were presented on the UAV P600 trajectory to point out the current YUAV axis directions. In addition, on each locus of the two objects, fifteen hollow circles were presented, which correspond to the arrows in the experiment. The fifteen time points were also marked and exhibited in Fig. 9, which shows the angle between the two object connection lines plus Π/2 and Xworld, and the angle curve between YUAV and Xworld. YUAV is the trajectory of the UAV and Xworld is the trajectory of the two objects.

As shown in Fig. 9, the maximum distance between the UAV trajectory line and the trajectory lines of the two yaks occurred at 21.086 s was − 0.342 rad. This was because that the positions of the two target objects (Xworld,Yworld) changed rapidly, and the yaw rate visual servo controller was unable to respond quickly to the abrupt change. During the later half period of the experiment, the controller always kept a distance around 0.26 rad, and at the final stage, it adjusted YUAV nearly perpendicular to the connection line. These results can demonstrate the performance of the visual servo controller to some extent.

## Discussion

Table 1 shows that 33.6% of PR-MOTA on YOLOV7 detection was achieved with the proposed tracker. It’s the highest value for all the online trackers (Online tracker –Real time processing tasks are required to track the position of objects in future frames through past and present frames) and comparable to the highest PR-MOTA for the batch IOU tracker. Note that the original algorithm of Deep SORT trained on appearance descriptors of our dataset was already able to achieve high PR-MOTA of 30.4% on YOLOV7 detection. Based on the improved algorithm, the proposed tracker could further enhance PR-MOTA of the original tracker by about 3.2%. Furthermore, a 4443.5 PR-FP decrease on the improved algorithm compared to Deep SORT was observed, revealing that PR-FP could be significantly reduced using the algorithm optimized in this paper. Meanwhile, the ID of our tracker significantly decreased compared to the ID of Deep SORT on the YOLOV7 detection. Moreover, in comparison with MOTDT and other methods based on Deep SORT improvement, the method proposed in this paper has the highest PR-MOTA value and the lowest PR-FP value, demonstrating better tracking performance. Results from experiments indicate that the improved algorithm could reduce false positive targets and enhance the tracking accuracy.

In summary, our method showed excellent tracking performance, which is reflected in three aspects: Detector, tracker and controller.

A powerful detector can improve the performance for target tracking. However, the performance of the target detection model in the livestock scene is easily influenced by many factors, such as illumination and occlusion. This is because of the poor generalization performance of the deep neural networks. Compared to R-CNN^63^, EB^64^ and other detection models, the YOLOv7^58^ detection model used as our detection component can more effectively alleviate the above problems, and thus improve the tracking performance.

In terms of target tracking, Alameer et al.^67^ used faster R-CNN and YOLOv2 as the detectors and Deep SORT as the tracker to overcome the problems of lighting variations and pig occlusion in commercial environments. In addition, they designed a deep learn-based pig posture and motor activity detection and Deep SORT tracking algorithm to analyze pig behavior changes in experimental pig houses under different greenhouse gas levels. These behavioral changes may be subtle indicators of declining health and welfare, which cannot be simply observed next to the farm fence. Their approaches were effective for detection and tracking behaviors. However, as video frames grow, due to dense overlap and occlusion, the pig ID errors occur during tracking. Therefore, their method is difficult to use for long-term tracking of different pig behaviors and cannot achieve accurate tracking of targets in dense occlusion scenes.

Tu et al.^68^ realized that the targets in pig videos are severely occluded and overlapped, and the lighting changes lead to incorrect switching of pig IDs during the tracking process, which reduces tracking quality. Therefore, they used the YOLO v5 detector to detect pigs and classify their behavior. In addition, it has developed an improved Deep SORT for pig behavior tracking and reducing error changes in pig IDs by improving trajectory processing (limiting target object ID growth specific to pig scenes) and data association (adding a second round of Intersection Over Union (IOU) matching to associate detection and tracking of mismatches). The methods can achieve pig behavior tracking with stable ID values under commercial conditions and provide scalable technical support for contactless automated pig monitoring.

But this method still has some limitations. In long-term tracking, the removal of detections with lower scores in the detector could result in erroneous deletion of some traces. The reason is that low-confidence detection boxes sometimes indicate the presence of objects, for example, occluded objects. Filtering out these objects causes irreversible errors in MOT and leads to non-negligible loss of detection and fragmentation trajectories. To solve this problem, we added a low-confidence trajectory filtering extension in Deep SORT, which provides an average confidence threshold for a two-stage filtration process. It reduces the false positive trajectory generated by Deep SORT, thereby mitigating the impact of unreliable detection on target tracking. Compared to the above methods, our method achieves better tracking and monitoring of animal herds in densely occluded scenes.

Wu et al.^69^ proposed a wheat counting method based on the UAV video multi-target tracking method by optimizing the YOLOv7 model and the Deep SORT algorithm. They used a modified deep ranking feature extractor to extract the feature information of wheat spikes and to re-identify the constructed dataset. Finally, the modified Deep SORT algorithm was used to calculate the number of different IDs appeared in the video, and then to develop an improved method based on the YOLOv7 and Deep SORT algorithms to calculate the number of small wheat ears in large fields. This method can efficiently detect, track and count wheat ears based on the ID values in the video. However, it is only applicable for the wheat field video with almost uniform speed, and its accuracy decreases in the complex scene of target movement.

In contrast, our method uses optical flow to compensate for the Kalman filter, which solves the problem of mismatched target bounding boxes predicted by the Kalman filter with inputs when the target detection in the current frame is complex. It compensates for the problem of inaccurate target prediction in nonlinear motion by the Kalman filter, thus achieving higher accuracy and can be well applied in the field of livestock monitoring.

In the area of UAV multi-object tracking, most studies focus only on the optimization of the algorithms^70–72^, and the following issue is neglected. Since the camera is fixed to the UAV, a rapid maneuver of the UAV causes dramatic changes in the view of the camera, resulting in the tracking failure or complete disappearance of the object from the view. In our study, this problem was taken into consideration. We used the servo control system to control the movement of the UAV to ensure the object within the view of the camera and the safe distance from the object, so as to assist the UAV in tracking multiple targets.

## Conclusion

A real-time target tracking system that can always keep the tracking targets within the view of a UAV camera is presented in this paper. The system considering the trade-off between accuracy and real-time performance, uses the YOLOv7 algorithm for target detection and the Deep SORT algorithm optimized in this work for target tracking. In addition, a visual servo controller for the UAV is designed to complete the automated tracking task. Based on the experimental results, the following conclusions are summarized:

In our improvement of the Deep SORT algorithm, the compensation of Kalman filter with optical flow for motion prediction is proposed to effectively solve the problem that the bounding box of the target predicted by the Kalman filter cannot match the input when the detection of the target in the current frame is complex. The embedded low confidence trajectory filtering method can significantly reduce false positive trajectories generated by Deep SORT, thus minimizing the impact of unreliable detections on target tracking. In addition, the visual servo controller can assist the UAV in multi-target tracking and identification of the yak group to ensure that the yak is within the view field of the camera with a safe distance.

Different multi-target tracking algorithms were compared in this study. The experimental results showed that among all online tracking algorithms, our tracking algorithm (PR-MOTA) has the highest accuracy, which is comparable to that of the batch processing IOU tracking algorithm. In comparison with the original Deep SORT, better results of PR-MOTA, PR-FP and IDs are achieved with our algorithm, which also performs better in terms of the PR-MOTA and PR-FP evaluation metrics compared to improved algorithms based on deep sorting such as MOTDT, proving the high effectiveness and advantage of the algorithm proposed in this study. In addition, this algorithm can be conveniently implanted into unmanned aerial vehicles with limited computing power for efficient real-time target tracking.

In the future research, the performance of our tracker will be further improved from the following aspects: Firstly, transfer learning will be used to train the YOLOv7 model to further enhance the accuracy of detection. Secondly, the IOU matching phase in the Deep SORT algorithm will be optimized to more effectively measure the match between the detection and prediction frames to improve tracking efficiency and robustness.

## Supplementary Information

Supplementary Information.

## Data Availability

Algorithm and dataset for this research can be found at the following data link (https://github.com/hardboy12/YOLOv7-DeepSORT.git).
