# Supplementary material for: An efficient visual servo tracker for herd monitoring by UAV
Source: Sci Rep. 2024 May 7;14:10463. doi: 10.1038/s41598-024-60445-4 (PMC11582714; doi:10.1038/s41598-024-60445-4)
Supplement: Supplementary file 1 — Supplementary Information. [file 41598_2024_60445_MOESM1_ESM.docx]

Appendix A.

**Hardware parameters:**

| **Hardware Name** | **Specific parameter settings** |
| --- | --- |
| 1.Rack | Aircraft type: quadcopter  Weight (approximately): 2.2kg (excluding battery, including load), 3.2kg (including battery, including load)  Diagonal wheelbase: 600mm  Power system: 6S FOC governor+4006 motor+370mm  Maximum payload of blade: 0.8kg (+pod+LiDAR)  Maximum takeoff weight: 4kg  Maximum flight time: 36min22s (high configuration, 4m flight altitude, outdoor 19 degrees)  Hover accuracy: 0.5m vertically and 1.5m horizontally  Wind resistance level: 6-7  Working environment: outdoor  Working temperature: 6 ℃ -40 ℃ |
| 2.Built in flight control | Flight control kernel: Based on Pixhawk FMUv5 deep optimization design, 90% compatible with PX4 FMUv5.  Integrated distribution board: DC-DC/2-ch 12V 3A/1-ch 5V 3A/4 * XT30 interface/100A solid-state relay  Main MCU chip: STM32F765VI/216MHz/2M program memory/512KB data memory  Gyroscope: ICM20689  Barometer: BMP388  MTD：AT24C64 |
| 3.External expansion platform | Power interface: 1 * XT30 battery voltage power supply port  I/O interface: built-in PX4IO-V2, supporting 8 * PWM interface/1 * RC interface (supporting Sbus, PPM, DSM)  External interface: 3 * UART (GH1.25 6Pin)/1 * CAN (GH1.25 4Pin)/Flight control USB connection (Type-C) |
| 4.On board computer | Name: Allspark  Model: AS1X  Weight: 213g  Size: 94 * 59 * 37 mm  Processor: NVIDIA Jetson NX  Memory：8G LPDDR4x @1600MHz  Emmc:16GB  SD card: 64GB (maximum support 128GB)  Display:Micro HDMI(1920*1080P)  Camera interface: MIPI Camera x2  Ethernet port: 1000Mbps (adapter)  WiFi：2.4G  USB interface:USB3.0 Port(Type A) x2、USB3.0 Port(Type C) x1、USB2.0 Port(Micro B)  x1(OTG)  GPIO:GPIO x5 (3.3V)  CAN:CAN x1(3.3V )  UART*3:UART x3(3.3V ,含Debug UART)  SPI:SPI x1(3.3V )  Overall power: 6~24W  Power input: 9-20V (recommended 12V, 2.5A)  Working environment temperature: -20 ℃ to 50 ℃ |
| 5.Battery | Model: 6S-10000mah lithium polymer battery  Effective capacity: 10000mAh  Battery weight: 1008g (approximately 1kg)  Operating voltage: 22.2V-26.1V  Minimum voltage: 21V (theoretical minimum 18V, actual minimum 22V)  Storage voltage: 23.1V (stored according to a single section voltage of 3.85V)  Battery interface: XT60  Battery type: LIHV lithium battery  Working temperature: 0 ℃ -60 ℃ |
| 6.Remote control | Model: H16 Number of channels: 16  Frequency range: 2.400~2.483 GHz  RF power: 20DB@CE / 23DB@FCC  Frequency hopping: New FHSS frequency hopping  Working voltage: 4.2V  Battery: 20000mAh  Endurance: 6-20 hours  Charging interface: Type-C  Upgrade: APP online upgrade  Size: 272 * 183 * 94mm  Weight: 1034g |
| 7.Communication link | Model: R16  Communication distance: 5-10km (nominal), 3km (measured in urban environment)  Bandwidth: 20Mbps  Number of channels: 16  RF power: 20DB@CE / 23DB@FCC  Working voltage: 7.2-72V  Interface: MIPI input * 1; HDMI input * 1; Network port * 1; BAT+SBUS * 1 Type-C * 1; TF card slot; Serial port * 2  Size: 76 * 59 * 11mm  Weight: 90g |
| 8.Searchlight camera | Model: MIPI ThreadBody  Video format: 1920* 1080@25fps  Light effect component: 8W high-power LED, 150 ° lens  Working voltage: 14-72V  Working current: 14mA (without turning on the light); 140mA (with lights on)  Size: 105 * 43 * 35mm  Weight: 55g |
| 9.Photoelectric pod | Model: Q10F Weight: 409g  Dimensions: length 105x width 91x height 98mm  Input voltage: 3s~4s  Video output: 360P (30fps), 480P (30fps, recommended for visual recognition) 720P (10fps), 1080P (5fps)  Camera performance: 1/3 inch 4 megapixel COMS SENSOR  Camera focal length: 10x optical zoom, F=4.9-49mm  Temperature working range: -10~45 degrees Celsius  Deviation pixel update rate: 50Hz  Minimum target size: 16x16 pixels  Target locking steady-state error:<5 pixels  Output interface: USB interface |
| 10.LiDAR | Model: LDS-50C  Laser ranging technology: TOF  Measurement radius: 0.15m~40m  Sampling speed: 9200 times/second  Distance measurement accuracy: 2-10cm (typical value 5cm)  Ranging resolution: 1cm  Scanning angle: 360 °  Scanning frequency: 7-15Hz (typical value 10Hz) |

Appendix B.

Low Confidence Track Filtering Algorithm

| Algorithm Low Confidence Track Filtering |
| --- |
| Input：Tentative tracks $T_{t}$；Tentative threshold $t_{tentative}$；  Average detection confidence threshold $t_{ave_{d}}$；Associated detection confidence $P_{t}$.  Output：Confirmed tracks $T_{c}$；Deleted tracks$T_{d}$.  1：for sequential frames do  2： for $t\in T_{t}$ do  3： if $t$ is new in $T_{t}$ then  4： hits = 0  5： total_prob = 0  6： hits = hits + 1  7： total_prob = total_prob +$P_{t}$  8： if $hits\geq t_{tentative}$ then  9： if $\frac{total\_prob}{hits}<t_{ave_{d}}$ then  10： $T_{d}=T_{d}\cup t$ and $T_{t}=T_{t}\backslash t$  11： else  12： $T_{c}=T_{c}\cup t$ and $T_{t}=T_{t}\backslash t$ |

Appendix C.

Terminology of the paper

| Terminology | Explanation |
| --- | --- |
| Agriculture 4.0 | The fourth agricultural revolution |
| IoT | Internet of things |
| UAV | Unmanned Aerial Vehicle |
| AI | Artificial Intelligence |
| KF | Kalman Filter |
| SSD | Single Shot MultiBox Detector |
| R-CNN | Regional Convolutional Neural Network |
| MOT | Multi-object tracking |
| td | Confidence threshold |
| $t_{ave_{d}}$ | Average confidence values |
| low td | Low confidence threshold |
|  | The lateral speed |
|  | The final velocity |
| $V_{World}$ | Expected velocity |
| PR-MOTA | Under different confidence thresholds, the values of precision and recall are obtained separately, and then the corresponding PR-MOTA can be obtained based on the different precision and recall. MOTA is the multi-target tracking accuracy , a key score for evaluating the tracking performance. |
| PR-MOTP | It is derived from the values of precision and recall under different confidence thresholds. MOTP is the multi-target tracking precision, which is a measure of the tracker's ability to estimate the target position. |
| PR-MT | PR-MT is originated from the values of precision and recall for different confidence thresholds. MT is the number of primary tracking traces that are successfully tracked during at least 80% of the target’s lifetime. |
| PR-ML | PR-ML is derived from the values of precision and recall under different confidence thresholds. ML is the quantity of the mostly lost tracks that are not successfully tracked during minimum 20% of the target's lifetime. |
| PR-FP | PR-FP is the total quantity of FPs. FP is the false positive |
| PR-FN | PR-FN means total quantity of FNs (target not met). FN is the Lost target |
| PR-IDs | PR-IDs is found under different confidence thresholds based on the values of precision and recall. IDs is the total number of identity switches(The ideal IDs in the tracking algorithm should be 0.). |
| PR-FM | With different confidence thresholds, the PR-FM is derived from the values of precision and recall. FM is the times of interruption for a track due to missing detection. |
| YUAV | The trajectory of the UAV |
| Xworld | The trajectory of the two objects |
| IOU | Intersection over Union |
